# Dental caries and associated factors in 3 to 5-year-old children in Zhejiang Province, China: an epidemiological survey

**DOI:** 10.1186/s12903-018-0698-9

**Published:** 2019-01-10

**Authors:** Na Zhou, Haihua Zhu, Yadong Chen, Wen Jiang, Xiaolong Lin, Yan Tu, Dingwan Chen, Hui Chen

**Affiliations:** 1 Department of Conservative Dentistry and Endodontics, Stomatological Hospital Affiliated to Medical College, Zhejiang University, 395 Yan’an Road, Hangzhou, China; 2 Hangzhou Medical College, 481 Binwen Road, Hangzhou, China

**Keywords:** Early child caries, Risk factors, Epidemiology, Body mass index, Breastfeeding duration

## Abstract

**Background:**

Dental caries in preschool children is prevalent worldwide, but data regarding its magnitude and associated factors were not available for preschool children in Zhejiang Province, China. This study examines the dental caries situation and its associated factors in Zhejiang Province.

**Methods:**

A total of 1591 children aged 3–5 years and their parents or caregivers were enrolled in this study. The condition of their teeth was assessed by three dental technicians qualified to WHO 2013 criteria. A structured questionnaire was completed by the children’s parents or caregivers. A logistic regression analysis was used to analyze the risk factors that may be associated with dental caries occurring among preschool children.

**Results:**

Caries prevalence (dmft> 0) of 3–5 year old children in Zhejiang Province was 70.4%. The mean decayed, missing and filled teeth (dmft) scores of the 3, 4 or 5 year old children surveyed were 2.96 ± 4.07, 4.42 ± 4.66, and 5.75 ± 5.19 respectively. The negative binomial regression model found that higher dental caries prevalence was found in children as age increased, with lower body mass index (BMI), with longer breastfeeding duration and with fewer hours of sleep.

**Conclusions:**

The dental caries prevalence and dmft score of 3–5-year-old children in Zhejiang Province was high, and it was associated with age, BMI, breastfeeding duration and hours slept.

**Electronic supplementary material:**

The online version of this article (10.1186/s12903-018-0698-9) contains supplementary material, which is available to authorized users.

## Background

Oral health is important for a child’s well-being and development. Dental caries, which remains a serious public issue worldwide, is one of the most common diseases, particularly in many developing countries, and in the past decades [1]. Although poor oral health is not life-threatening, it has a deleterious impact on other diseases, and may cause dental pain, sleep disturbance, reduced weight and height development, reduced speech development, poor self-esteem or altered quality of life, and the likelihood of caries increasing in secondary dentition [2].

Early child caries (ECC) is defined as the presence of decayed, missing, and filled tooth surfaces in any deciduous dentition occurring in a child 71 months or younger [2]. Previous research has shown that the global prevalence of ECC remains high, up to a level of 70% [3]. It has become a global burden on social and economic health. The factors that are currently associated with ECC are the life time of dentition [4], oral health behavior (e.g., tooth brushing and using fluoridated toothpaste) [5], feeding practices (e.g., breast feeding practice, night bottle-feeding) [5–7], nutritional habits (obesity, sugar intake, dietary habits) [8], socio-economic status (e.g., family annual income, maternal education) and geographic location [2, 9]. To the best of our knowledge, previous studies do not provide enough evidence to demonstrate that feeding practice, nutritional habits, socio-economic status or geographic locations are causative factors for ECC [4–9]. Therefore, it is important to verify children with dental caries and determine the risk indicators of ECC, because studying the prevalence of ECC and its associated indicators in a community assists in determining its public health importance and the ways to control it.

Zhejiang Province is located in eastern coastal China. It has more than 55 million permanent residents, features a booming economy and high population density. The present study was designed to determine the prevalence rate of ECC and its associated biological, diet or health-related variable factors among preschool children aged 3–5 years in Zhejiang Province.

## Methods

This cross-sectional study was conducted from January to June 2016 in Zhejiang Province, which was one component of the Fourth National Oral Health Survey in China. The Ethics Committee of the Chinese Stomatological Association approved this research (NO.2014–003).

The target population was 3–5 year old residents in Zhejiang province who had been living in the sampling area for more than six months. Age was calculated based on the survey month.

The sample size was calculated according to the following formula:

$$ n= deff\frac{\upmu_a{}^2p\left(1-p\right)}{\delta^2} $$

In this formula, *n* was the sample size, the design effect *deff* was set at 4.5, μ was the level of confidence, *p* was the dental caries prevalence set at 66.0% (according to the Third National Oral Health Survey), and δ was the margin of error. The non-response rate was 20%, so the minimum required sample size was 1296 in Zhejiang Province.

A multistage, stratified and random cluster sampling procedure was used to select the representative study sample. Population data was obtained from the 2010 census conducted by the National Statics Bureau. First, two districts and two counties of Zhejiang Province were randomly selected by the probability-proportional-to-size sampling (PPS) method. Second, three public or private kindergartens in each district or county were chosen using the PPS method. Third, 138 preschool children aged 3, 4, and 5 years in the chosen kindergartens were recruited using a quota sampling method.

Before the survey, three examiners were trained by a qualified examiner in theoretical knowledge and clinical practice. Then, each examiner enrolled three participants to calibrate the examinations with the qualified examiner. The mean Kappa value used to determine the inter-examiner reproductivity was > 0.85. In addition, inter-examiner reproducibility was checked by random re-examination of 5% of the samples, and the Kappa value was recorded for each oral survey in the kindergartens.

All kindergartens enrolled in this research received information and agreed to participate. Written informed consent was obtained from parents or guardians for minors to participate in this study. A clinical oral examination of children, accompanied by a questionnaire for parents or caregivers, was done to assess ECC.

Three trained dentists performed the oral examination in the chosen kindergartens. The oral health survey was performed in accordance with the WHO guidelines for assessment of dental status [10]. Caries was diagnosed at cavitation level, which was confirmed by a ball-end Community Periodontal Index (CPI) probe. When the oral examination was finished, a leaflet explaining the child’s oral health and recommended treatment protocols, based on the examination results, was sent to the child’ s parents or guardians.

Each child’s height (kg) and weight (m) were measured before the oral examination. Body height and weight were measured with the child bare-footed. Body height was measured using a stadiometer and body weight was determined by a portable digital scale. The formula: BMI = mass (Kg)/[height (m)] ^2^ was calculated to give body mass index (BMI). The cut off points of BMI values were the 25, 50, 75 and 100% percentiles, and BMI value was divided into four categories: 0–25% percentile, 25–50% percentile, 50–75% percentile, and 75–100% percentile.

Two days before the physical examination, the informed consent forms were given to each of the enrolled children’s parents or caregivers. One day before the physical examination, structured questionnaires were completed by the parents or caregivers, who were interviewed face-to-face and one-on-one by trained interviewers. Then the interviewers verified the completeness of the answering of the questionnaires after collecting them. The questionnaires contained questions on subjects’ dietary intake, oral health behavior, infant feeding practice, maternal education, and calcium intake during pregnancy. An additional file shows this in more detail [see Additional file 1].

To minimize the risk of data entry errors, the data was entered twice. The statistical software SPSS 16.0 (SPSS, Inc., Chicago, IL, USA) was used to assess the data. A trend chi-square test was performed to test differences for dental caries prevalence among different groups according to the variables studied. Mann-Whitney U tests or Kruskal-Wallis one-way analysis was used to analyze decayed, missing and filled teeth (dmft) scores, decayed teeth (dt) scores, missing teeth (mt) scores, and filled teeth (ft) scores using various categories of variables.

A logistics regression analysis was used to explore risk indicators which might affect dental caries experiences in preschool children. To inquire into the risk factors which may be associated with caries prevalence (dmft>0), variables with *P*-value < 0.10 in the chi-square test were included in the negative binomial regression model. This minimized the influence of potentially irrelevant variables and prevented the overloading of important variables. At each step of a backwards stepwise procedure, insignificant variables (*P* > 0.05) were removed, until only the variables that showed significant association (*P* < 0.05) remained.

## Results

A total of 1656 preschool children were invited; 1620 children’s parents or caregivers signed the ethics consent forms and agreed to participate the survey; 29 samples with missing information were excluded in the data entry process; so a total of 1591 samples were included in the final data analysis.

The response rate was 97.8% (1620/1656) for 3 to 5-year olds investigated in this study. Of the total sample, 51.6% were boys and 48.4% were girls. Caries prevalence (dmft≥0) was 70.4%, and mean dmft was 4.34 ± 4.78; 98.1% of decayed teeth had not been treated.

The characteristics and the results of the survey are shown in Table 1.

**Table 1:** Characteristics of participants and the results of the survey

Variables	n (%) dmft = 0	n (%) dmft> 0	dmft (mean ± SD)	dt	mt	ft
General characteristics of the children						
Total (*n* = 1591)	471 (29.6)	1120 (70.4)	4.34 ± 4.78	4.26	< 0.01	0.08
Sex						
Male (*n* = 821)	243 (29.6)	578 (70.4)	4.28 ± 4.67	4.2	< 0.01	0.08
Female (*n* = 770)	228 (29.6)	542 (70.4)	4.40 ± 4.89	4.32	< 0.01	0.08
Age (years)						
3 (*n* = 531)	213 (40.1)	318 (59.9)**	2.96 ± 4.07**	2.92**	< 0.01	0.04*
4 (*n* = 571)	150 (26.3)	421 (73.7)	4.42 ± 4.66	4.35	< 0.01	0.07
5 (*n* = 489)	108 (22.1)	381 (77.9)	5.75 ± 5.19	5.62	< 0.01	0.13
Biological and social-economic variables						
BMI						
0–25% percentile(11.0–14.31)	91 (22.8)	308 (77.2)**	4.85 ± 4.96*	4.77*	< 0.01	0.08
(*n* = 399)						
25–50% percentile(14.31–15.29)	107 (26.4)	298 (73.6)	4.44 ± 4.78	4.35	< 0.01	0.09
(*n* = 405)						
50–75% percentile (15.29–16.54)	95 (28.6)	237 (71.4)	4.31 ± 4.76	4.27	< 0.01	0.04
(*n* = 332)						
75–100% percentile(16.54–23.61)	178 (39.1)	277 (60.9)	3.82 ± 4.59	3.73	< 0.01	0.1
(*n* = 455)						
Only child in the family						
Yes (*n* = 1120)	386 (34.5)	734 (65.5)	4.26 ± 4.77	4.17	< 0.01	0.09
No (*n* = 471)	153 (32.5)	318 (67.5)	4.50 ± 4.78	4.44	< 0.01	0.06
Mother’s education level						
Primary or below (*n* = 127)	41 (32.3)	86 (67.7)	4.06 ± 4.49	3.97	< 0.01	0.09
Junior high school (n = 571)	159 (27.8)	412 (72.2)	4.50 ± 4.82	4.43	< 0.01	0.06
Senior high school (*n* = 470)	147 (31.3)	323 (68.7)	4.42 ± 5.02	4.34	< 0.01	0.08
Matriculation or above (*n* = 423)	124 (29.3)	299 (70.7)	4.12 ± 4.53	4.03	< 0.01	0.09
Diet-related variables						
Sugary food consumption frequency						
Sweets or sugary snacks						
Twice a week or more (*n* = 1044)	321 (30.7)	723 (69.3)	4.31 ± 4.73	4.22	< 0.01	0.08
Once a week (*n* = 219)	67 (30.6)	152 (69.4)	4.64 ± 527	4.58	< 0.01	0.06
Seldom or never (*n* = 328)	83 (25.3)	245 (74.7)	4.25 ± 4.59	4.17	< 0.01	0.08
Sugary beverage						
Twice a week or more (*n* = 540)	172 (31.9)	368 (68.1)	4.27 ± 4.87	4.22	< 0.01	0.05
Once a week (*n* = 277)	71 (25.6)	206 (74.4)	4.63 ± 4.69	4.58	< 0.01	0.05
Seldom or never (*n* = 774)	228 (29.5)	546 (70.5)	4.28 ± 4.75	4.17	< 0.01	0.11
Sugary dairy foodstuffs						
Twice a week or more (*n* = 943)	283 (30.0)	660 (70.0)	4.37 ± 4.87	4.29	< 0.01	0.08
Once a week (*n* = 176)	44 (25.0)	132 (75.0)	4.43 ± 4.30	4.38	< 0.01	0.06
Seldom or never (*n* = 472)	144 (30.5)	328 (69.5)	4.25 ± 4.77	4.17	< 0.01	0.08
Cooked food consumption frequency						
Seafood						
Twice a week or more (*n* = 490)	141 (28.8)	349 (71.2)	4.45 ± 4.88*	4.36	< 0.01	0.10**
Once a week (*n* = 304)	76 (25.0)	228 (75.0)	4.90 ± 4.96	4.73	< 0.01	0.17
Seldom or never (*n* = 797)	254 (31.9)	543 (68.1)	4.06 ± 4.62	4.02	< 0.01	0.03
Meat						
Twice a week or more (*n* = 1292)	385 (29.8)	907 (70.2)	4.21 ± 4.73	4.12*	< 0.01	0.09
Once a week (*n* = 124)	37 (29.8)	87 (70.2)	4.75 ± 5.06	4.65	< 0.01	0.10
Seldom or never (*n* = 175)	49 (28.0)	126 (72.0)	5.01 ± 4.90	4.99	< 0.01	0.02
Egg & soy foods						
Twice a week or more (*n* = 1206)	358 (29.7)	848 (70.3)	4.31 ± 4.76	4.23	< 0.01	0.08
Once a week (*n* = 194)	59 (30.4)	135 (69.6)	4.30 ± 4.88	4.18	< 0.01	0.12
Seldom or never (*n* = 191)	54 (28.3)	137 (71.7)	4.59 ± 4.82	4.55	< 0.01	0.04
Vegetables & fruit						
Twice a week or more (*n* = 1453)	441 (30.4)	1012 (69.6)	4.25 ± 4.75*	4.17*	< 0.01	0.08
Once a week (*n* = 52)	14 (26.9)	38 (73.1)	4.50 ± 4.81	4.37	< 0.01	0.13
Seldom or never (*n* = 86)	16 (18.6)	70 (81.4)	5.85 ± 4.96	5.80	< 0.01	0.05
Yoghourt consumption frequency						
Twice a week or more (*n* = 612)	198 (32.4)	414 (67.6)	4.05 ± 4.74	3.95	< 0.01	0.10
Once a week (*n* = 526)	150 (28.5)	376 (71.5)	4.51 ± 4.91	4.43	< 0.01	0.08
Seldom or never (*n* = 453)	123 (27.2)	330 (72.8)	4.53 ± 4.66	4.48	< 0.01	0.05
Milk consumption						
0-100 ml/day (*n* = 518)	160 (30.9)	358 (69.1)	4.33 ± 4.86	4.25	< 0.01	0.09
100-200 ml/day (*n* = 734)	202 (27.5)	532 (72.5)	4.42 ± 4.75	4.35	< 0.01	0.07
200-400 ml/day (*n* = 339)	109 (32.2)	230 (67.8)	4.19 ± 4.73	4.1	< 0.01	0.09
Health-related variables						
Tooth brushing						
Often (*n* = 593)	183 (30.86)	410 (69.14)	4.22 ± 4.79	4.13	< 0.01	0.08
Seldom or never (*n* = 998)	288 (28.86)	710 (71.14)	4.41 ± 4.77	4.33	< 0.01	0.08
Toothpaste use						
Yes (*n* = 578)	180 (31.1)	398 (68.9)	4.22 ± 4.81	4.14	< 0.01	0.07
No (*n* = 1013)	291 (28.7)	722 (71.3)	4.41 ± 4.76	1.33	< 0.01	0.08
Fluoride toothpaste use						
Yes (*n* = 95)	30 (31.6)	65 (68.4)	3.75 ± 4.05	3.71	< 0.01	0.04
No (*n* = 1496)	441 (29.5)	1055 (70.5)	4.38 ± 4.82	4.3	< 0.01	0.08
Infant breastfeeding duration						
0–6 months (*n* = 465)	148 (31.8)	317 (68.2)*	3.93 ± 4.53*	3.85*	< 0.01	0.08
7–12 months (*n* = 684)	213 (31.1)	471 (68.9)	4.28 ± 4.84	4.18	< 0.01	0.10
13–18 months (*n* = 301)	81 (26.9)	220 (73.1)	4.66 ± 4.93	4.6	< 0.01	0.06
≥ 18 months (*n* = 141)	29 (20.6)	112 (79.4)	5.32 ± 4.80	5.28	< 0.01	0.04
Mother’s calcium intake during pregnancy						
Yes (*n* = 1215)	359 (29.5)	856 (70.5)	4.40 ± 4.85	4.33	< 0.01	0.08
No (*n* = 240)	69 (28.7)	171 (71.3)	4.13 ± 4.69	4.04	< 0.01	0.10
Uncertainty (*n* = 136)	43 (31.6)	93 (68.4)	4.13 ± 4.27	4.07	< 0.01	0.06
Infant Vitamin D intake						
Never (*n* = 229)	72 (31.4)	157 (68.6)	3.66 ± 4.15*	3.63*	< 0.01	0.03
Less than one year (*n* = 997)	274 (27.5)	723 (72.5)	4.55 ± 4.83	4.47	< 0.01	0.08
More than one year (*n* = 365)	125 (34.2)	240 (65.8)	4.19 ± 4.96	4.08	< 0.01	0.11
Time of falling asleep						
Before 9 pm (*n* = 444)	138 (31.08)	306 (68.92)	3.97 ± 4.48	3.91	< 0.01	0.06
9–11 pm (*n* = 988)	282 (28.54)	706 (71.46)	4.48 ± 4.87	4.37	< 0.01	0.09
After 11 pm (*n* = 38)	10 (26.32)	28 (73.68)	5.03 ± 5.17	5.00	< 0.01	0.03
Irregular (n = 121)	41 (33.88)	80 (66.12)	4.50 ± 4.95	4.4	< 0.01	0.10
Hours of sleep						
≥ 12 h (*n* = 130)	43 (33.1)	87 (66.9)**	3.37 ± 4.30*	3.30*	< 0.01	0.07
10–12 h (*n* = 912)	299 (32.8)	613 (67.2)	4.24 ± 4.85	4.16	< 0.01	0.08
≤ 10 h (*n* = 549)	129 (23.5)	420 (76.5)	4.74 ± 4.72	4.66	< 0.01	0.08
Family smoking environment						
Yes (*n* = 1049)	312 (29.7)	737 (70.3)	4.38 ± 4.84	4.32	< 0.01	0.06
No (*n* = 542)	159 (29.3)	383 (70.7)	4.26 ± 4.66	4.15	< 0.01	0.11

*p < 0.05

** *p* < 0.001

Five-year-old children had more dental caries (dmft = 5.75, dt = 5.62) compared to 4-year-old (dmft = 4.42, dt = 4.35) and 3-year-old (dmft = 2.96, dt = 2.92) children (*P* < 0.001). The dental caries prevalence (dmft> 0) increased with age (*p* < 0.001).

BMI differences were found in the prevalence of dental caries (*P* < 0.001), dmft score, dt score (*P* < 0.05). Children with higher BMI values had lower dmft scores, dt scores and lower caries prevalence (*P* < 0.05).

For the diet-related factors, no association was found between dental caries and sugary food consumption frequency, milk or yoghurt consumption (*P* > 0.05). Children eating seafood at a median frequency (once a week) had higher dmft scores (*P* < 0.05), children eating more meat had lower dt scores (*P* < 0.05), children eating more vegetables and fruit had lower dmft and dt scores (*P* < 0.05).

For the health-related variables, infant breastfeeding duration was associated with caries prevalence, dmft and dt scores (*P* < 0.05). Children who never took Vitamin D in infancy had lower dmft and dt scores (*P* < 0.05). Children sleeping for longer hours had lower caries prevalence (*P* < 0.001), dmft and dt scores (*P* < 0.05). In addition, no association was found between tooth decay occurrence and frequency of tooth brushing, the use of toothpaste or fluoride toothpaste, mother’s calcium intake during pregnancy, time before falling sleep or family smoking circumstances (*P* > 0.05).

Table 2 shows the analysis results for the related risk factors and dental caries prevalence using a backward logistic regression model. Six variables with *p*-value < 0.10 (age, BMI, sea food consumption frequency, vegetables and fruit consumption frequency, breast feeding duration and sleeping hours) were included in the negative binomial regression model. Five risk factors were presented in the final model results: greater age, lower BMI, sea food consumption once a week, longer breastfeeding duration and fewer hours of sleep. These were indicators of preschool children’s dental caries (*P* < 0.05).

**Table 2:** Results of negative regression for dmft> 0 among the surveyed preschool children in Zhejiang Province

	IRR^a^ (95% CI^b^)	Wald (Χ^2^)	*P*
Age groups			
3-year-old	0.44 (0.34,0.59)	31.47	<0.001
4-year-old	0.78 (0.58,1.04)	2.84	0.09
5-year-old	–––	–––	–––
BMI			
0–25% percentile	2.20 (1.62,3.00)	25.14	<0.001
(11.0–14.31)			
25–50% percentile	1.85 (1.37,2.49)	16.11	<0.001
(14.31–15.29)			
50–75% percentile	1.86 (1.36,2.54)	14.97	<0.001
(15.29–16.54)			
75–100% percentile	–––	–––	–––
(16.54–23.61)			
Seafood consumption frequency			
Twice a week or more	1.26 (0.97,1.63)	3.07	0.08
Once a week	1.48 (1.08,2.02)	6.09	0.01
Seldom or never	–––	–––	–––
Infant breastfeeding duration			
0–6 months	0.55 (0.34,0.88)	6.25	0.01
7–12 months	0.59 (0.37,0.93)	5.22	0.02
13–18 months	0.80 (0.49,1.32)	0.78	0.38
≧18 months	–––	–––	–––
Sleeping hours			
≥ 12 h	0.66 (0.43,1.01)	3.67	0.06
10–12 h	0.66 (0.51,0.85)	10.70	0.001
≤ 10 h	–––	–––	–––

^a^Incidence rate ratio

^b^95% confidence interval

## Discussion

In Zhejiang Province in China, children more than 3 years old are sent for education to the nearest kindergarten, based on place of residence. A multistage sampling method was used in this study. 3 to 5-year-old children in public or private kindergartens were enrolled. With the help of local institutions such as Centers for Disease Control and Prevention (CDC) and the close co-operation of kindergarten teachers, the response rate was satisfactory.

Out of a total of 1591 3–5 year old children, 1120 children were affected with dental caries, showing an overall prevalence of 70.4%: 98.1% of decayed teeth found were not treated. Among the three age groups, 3-year-olds showed the lowest prevalence, at 59.9% and dmft 2.96, while 5-year-olds showed the highest prevalence, at 77.9% and dmft 5.75. A significant relationship was found between age and children’s dental caries prevalence (Table 1 and Table 2). These differences are probably because caries occurrence measures the continuous and cumulative effects of dental caries in the life-time of a particular dentition. Therefore, older children are likely to have a higher dmft score than younger ones.

The average prevalence and dmft of dental caries in 5-year-olds in China in year 2005 were 66% and 3.5 respectively, and about 97% of the decayed teeth in children aged five went untreated [11]. The prevalence rate for ECC remained high and rose even higher in the last decade, from 2005 to 2016. Zhejiang Province is economically prosperous compared to central and western China, its per capita gross domestic product (GDP) was over 10,000 USD by 2016, which was similar to Turkey and Brazil. An oral epidemiological survey on five-year-old children conducted in 2015 in Turkey showed that the caries prevalence was 84.1%, and the dmft was 4.41 [12]. A cross-sectional study extracted from the database of a 2010 Brazilian Health Survey found that more than half of 5-year old children had dental caries (54.1%) and the mean dmft was 2.42 [13]. The dental caries experience of Zhejiang Province was equivalent to Turkey but worse than Brazil. The caries situation in China shows characteristics typical of developing countries. With the development of the economy, more ultra-processed food rich in sugar and saturated fat is produced industrially, and people consume more cariogenic food, while oral health education hasn’t been conducted to match the continuous economic growth [14].

Despite awareness of poor oral health, the attitude toward oral health care was negative, as Table 1 shows. Only 37.3% of 3–5 year olds regularly brushed their teeth. Among children who regularly brushed their teeth, only 16.4% (30/183) used fluoride toothpaste. Though its GDP has increased rapidly in recent decades, and the rapid economic growth has changed life-styles, a large part of the population still lacks oral health knowledge and education. Since primary teeth will be replaced by permanent teeth, oral health care for children is not thought to be important.

Research into the relationship between dmft and BMI value in children has been controversial. Some studies investigating this relationship showed a positive correlation [15–17], yet some studies found an inverse relationship [18–20]. Some studies showed no effect of BMI values on dental caries [21, 22], and one study found a u-shaped association [23]. Body mass index changes substantially with age [24]. In this research, the subjects investigated were 36–60 months old, so a cut-off point based on age to define child obesity is ambiguous [24]. Therefore, in this study we explored the relationship between tooth decay and BMI value, and found that children with higher BMI values had significantly lower tooth decay prevalence and dmft scores. Children with lower BMI values (0–25% percentile, 25–50% percentile, 50–75% percentile) were more likely to have caries compared to those with higher BMI values (75–100% percentile) (Incidence rate ration (IRR) = 2.20, 1.85, 1.86 respectively, *P* < 0.001). Dietary habits may be the reason for this inverse association. Previous studies reasoned that overweight children might consume less sugary food but more fatty acids, thus fewer caries were developed while the children remained overweight [25, 26]. This was consistent with the finding that children who ate the least vegetables and fruit had the lowest dmft scores (Table 1).

This study also verified the influence of toothbrushing habits, breastfeeding practices, sleeping habits, and environmental and socio-cultural backgrounds on the occurrence of ECC. Our results concur with other studies: the prolonged breastfeeding may be a risk factor for a higher prevalence of dental caries [27–29]. We do not have a definite explanation for the mechanisms of our results. Methodological disparities, such as the timing of outcome assessment, breastfeeding exclusivity, and the lack of control over important confounding factors may have led to the contradictory results in previous studies. A correlation between caries experience and sleeping duration was found in this study, which agreed with a new published paper [30]. Shortness of sleep may lead to disrupted circadian rhythms, which reduced salivary flow rate, and resulted in an increasing risk of caries [31].

It is suggested that socio-economic status affects parents’ knowledge and attitudes towards child care [32]. Previous studies found a relationship between social gradients and oral health in children [33–35]. In the questionnaire for this research, about one in five or six parents or caregivers refused to answer the questions about family income, so valid family income information for the sample was not collected. However, maternal education is one popular way to measure socio-economic position in epidemiological studies [36]. No association was found between maternal education and ECC in this research. Similarly, it was found that ECC was not associated with a family smoking environment, or with the number of siblings.

Vitamin D and calcium are essential nutrients for bone health [37]. Infant birth weight was positively related to maternal intake of calcium [38]. The development of deciduous teeth begins in the embryonic stage. Also suggested was the importance of calcium intake during pregnancy and the role played by vitamin D for infants under the age of two in preventing caries later in childhood. No association was found between ECC and pregnancy calcium intake or infant vitamin D intake in this study, in contrast to previous studies [39, 40]. In this research, the co-factors of food variety and daily food consumption were not controlled. Serum calcium concentration was undetectable due to the limitation of study design. It is inappropriate to draw a relationship between ECC and pregnancy calcium intake or infant vitamin D intake.

This study has a few limitations. Either a recall or a response bias may be induced when the method of self-administration by parents was used in this retrospective study, and valid information about family income was not collected. A family’s education background or social status may determine the health beliefs or dental healthcare utilization, so the dental status of preschool children may vary according to differing family background, dental healthcare education of the community, dental health facilities utilization of the city, etc. Though the sample size is larger than the required sample size, there in total 90 districts and counties in Zhejiang province, so the four districts or counties randomly selected with a PPS method may not completely represent Zhejiang province overall.

Moreover, those not providing informed consent or those submitting incomplete questionnaires were also excluded, though this number is small. However, we could identify the correlation between risk factors and caries using cross-sectional study. A longitudinal study will better illustrate the predictors of ECC.

## Conclusions

This study finds a high ECC prevalence among children aged 3–5 years old living in Zhejiang Province, China. It shows a trend of an increase in ECC compared to other regions in China in 2005, and this despite the province’s undoubted increase in overall wealth in the past ten years. Age, BMI, seafood consumption frequency, infant breastfeeding duration and hours of sleep were the significant risk factors for dental caries’ occurrence in 3 to 5-year-old preschool children in Zhejiang, China. As children often follow their parents’ oral health behavior, interventions should be designed to educate families and change their attitudes toward oral health care.

## Additional file

Additional file 1: The Questionnaire of Fourth National Oral Health Survey and Zhejiang Provincial Oral Health Survey (Version for Children’s Guardians). The questionnaire of the oral health survey, the version for children’s guardians. (DOCX 42 kb)

## Abbreviations

BMI: Body mass index
CDC: Centers for disease control and prevention
CI: Confidence ratio
CPI: Community periodontal index
dmft: Decayed, missing and filled teeth
dt: Decayed teeth
ECC: Early child caries
ft.: Filled teeth
GDP: Gross domestic product
IRR: Incidence rate ratio
PPS: probability-proportional-to-size sampling
