# Supplementary material for: Dental caries and associated factors in 3 to 5-year-old children in Zhejiang Province, China: an epidemiological survey
Source: BMC Oral Health. 2019 Jan 10;19:9. doi: 10.1186/s12903-018-0698-9 (PMC6329098; doi:10.1186/s12903-018-0698-9)
Supplement: Supplementary file 1 — The Questionnaire of Fourth National Oral Health Survey and Zhejiang Provincial Oral Health Survey (Version for Children’s Guardians). The questionnaire of the oral health survey, the version for children’s guardians. (DOCX 42 kb) [file 12903_2018_698_MOESM1_ESM.docx]

**The Questionnaire of Fourth National Oral Health Survey and Zhejiang Provincial Oral Health Survey (Version for Children’s Guardians)**

Subject’s ID: Name of the subject:

Subject’s birthdate (mm/dd/yyyy): Gender (F or M):

Date of survey: No. of the interviewer:

Please draw a “√” before the corresponding option, only one answer for all the questions.

1. You are child’s?

1) Father 2) Mother

3) Grandfather 4) Grandmother

2. How often does your child have the food and beverage below?

6

≧twice

/day

5

once

/day

4

2-6 times

/week

3

once

/week

1

seldom

/never

2

1-3 times

/month

1) Sugary snacks (cookies, cakes, bread) and candies

2) Sugary beverage (sugary water,

sodas, and fruit juice with added sugar)

3) Sugary milk, yoghurt, tea, soya milk

and coffee

3. How often does your child have cooked food below?

2

1-3 times

/month

6

≧twice

/day

5

once

/day

4

2-6 times

/week

3

once

/week

1

seldom

/never

1. Seafood
2. Meat (including pork, beef,

chicken and lamb)

1. Eggs and soy food
2. Vegetables and fruit

4. How often does your child brush his/her teeth? (Those who choose Answer 2 please don’t answer Questions 5 -6)

1) Often 2) Seldom/never

5. Does your child use toothpaste while brushing his/her teeth? (Those who choose Answer 2 please don’t answer Questions 6)

1) Yes 2) No

6. Does your child use fluoride toothpaste?

1) Yes 2) No

7. Up to now, what is the child’s mother’s final education?

1) Primary or below 2) Junior high school

3) Senior high school 4) Matriculation or above

8. In the past 12 months, what is the total income of your family? *10,000 RMB/ year. (Please fill in a round number, those who decline to answer please fill with “N”.)

9. Is the investigated child the only one child in the family?

1) Yes 2) No

10. What is the time of your child to fall asleep?

1) Before 9 pm 2) 9-11 pm

3) After 11 pm 4) Irregular

11. How long does your child sleep every day? (including day time and night time)

1)12-14 hours 2)10-12 hours

3)8-10 hours 4)Less than 8 hours

13. How old was the child when the breastfeeding stopped?

1)0-3 months 2)4-6 months

3)7-9 months 4)10-12 months

5)13-18 months 6)19-24 months

7)Older than 24 months

14. How much milk does your child have every day?

1)Less than 100 mL 2)100-200 mL

3)200-400 mL 4)More than 400 mL

15. How often does your child have yogurt?

1)≧twice/day 2)once/day

3) 2-6 times/week 4)once/week

5)1-3 times/month 6) seldom/never

16. Did the child’s mother take calcium tablets during pregnancy?

1) Yes 2) No

3) Not sure

17. Did the child take Vitamin D after birth?

1)Never 2)Yes, less than 6 months

3)Yes, 6-12 months 4)Yes, 13-24 months

5)Yes, >24 months

18. Does anyone smoke and live together with the child?

1)Yes 2)No
